# Supplementary material for: Analysis of the Genomes and Adaptive Traits of Skermanella cutis sp. nov., a Human Skin Isolate, and the Type Strains Skermanella rosea and Skermanella mucosa
Source: Microorganisms. 2025 Jan 6;13(1):94. doi: 10.3390/microorganisms13010094 (PMC11767975; doi:10.3390/microorganisms13010094)
Supplement: Supplementary file 1 [file microorganisms-13-00094-s001.zip › microorganisms-3386424-supplementary.pdf]

Supplementary

Analysis of the Genomes and Adaptive Traits of *Skermanella cutis* sp. nov., a Human Skin Isolate, and the Type Strains *Skermanella rosea* and *Skermanella mucosa*

Yujin Choi, Munkhtsatsral Ganzorig and Kyoung Lee*

Department of Bio Health Science, Changwon National University, Changwon 51140, Gyeongnam, Republic of Korea

* Correspondence: kyounglee@changwon.ac.kr; Tel.: +82-055-213-3486

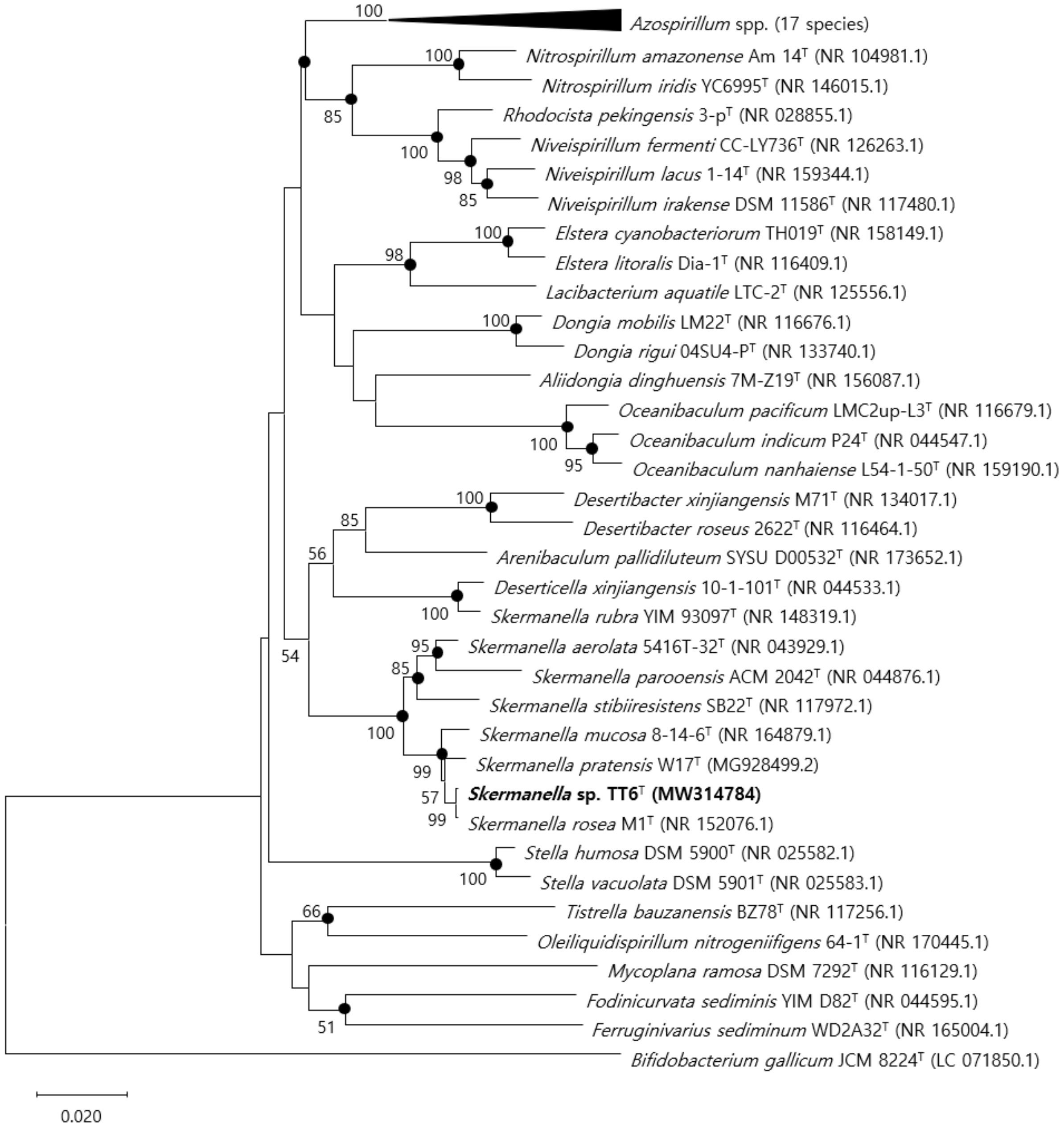

Figure S1. Neighbour-joining tree based on 16S rRNA gene sequences. The phylogenetic relationships between TT6^{T} (bold) and other type strains with >90% 16S rRNA gene sequence similarity to TT6^{T} are shown. The 16S rRNA NCBI GenBank accession number for each strain is shown in brackets. *Bifidobacterium gallicum* JCM 8224^{T} is used as the outgroup. Numbers at nodes represent bootstrap values (based on 1,000 resamplings) as percentages. The distance scale of 0.020 corresponds to a 2% dissimilarity between two sequences.

(A) **(B)**

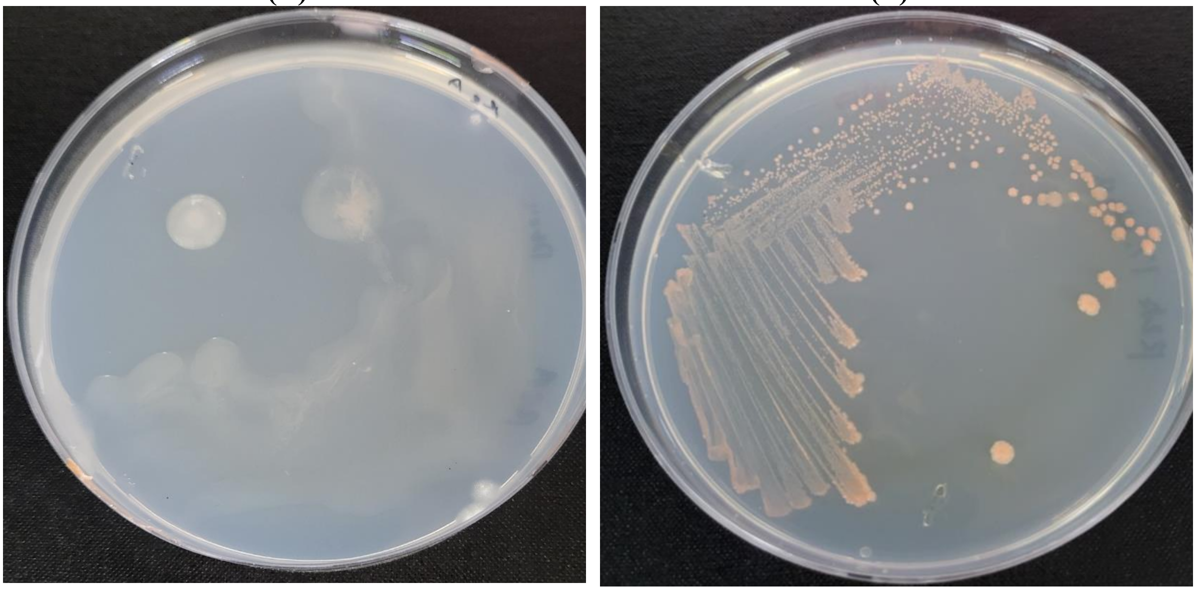

Figure S2. Colonies of strain TT6^T formed on R2A agar (A) and R2A agar with 1% NaCl (B). The cells were incubated for 3 days at 30 ℃.

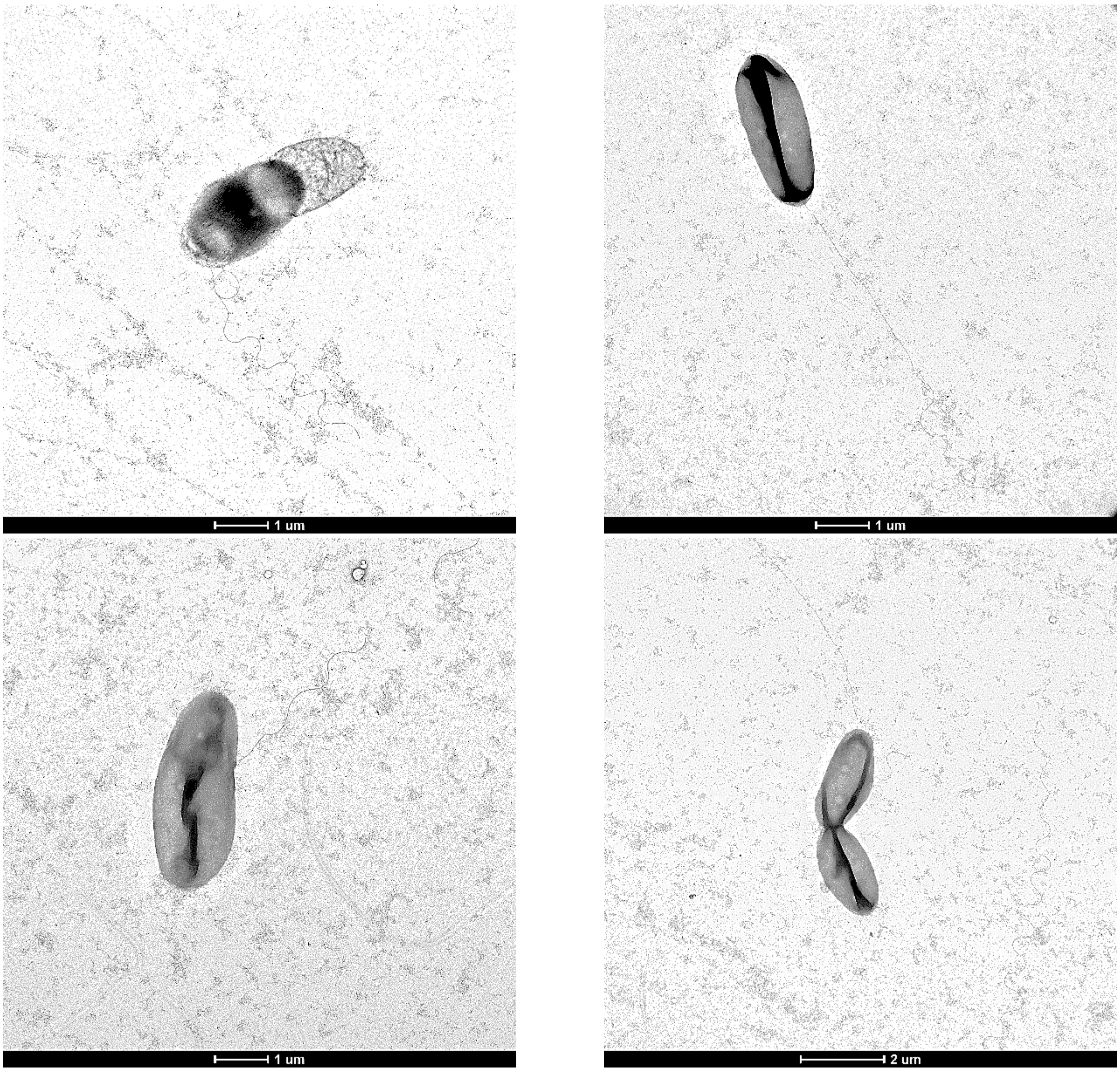

Figure S3. Transmission electron micrographs showing the general morphology of negatively stained TT6^{T} cells.

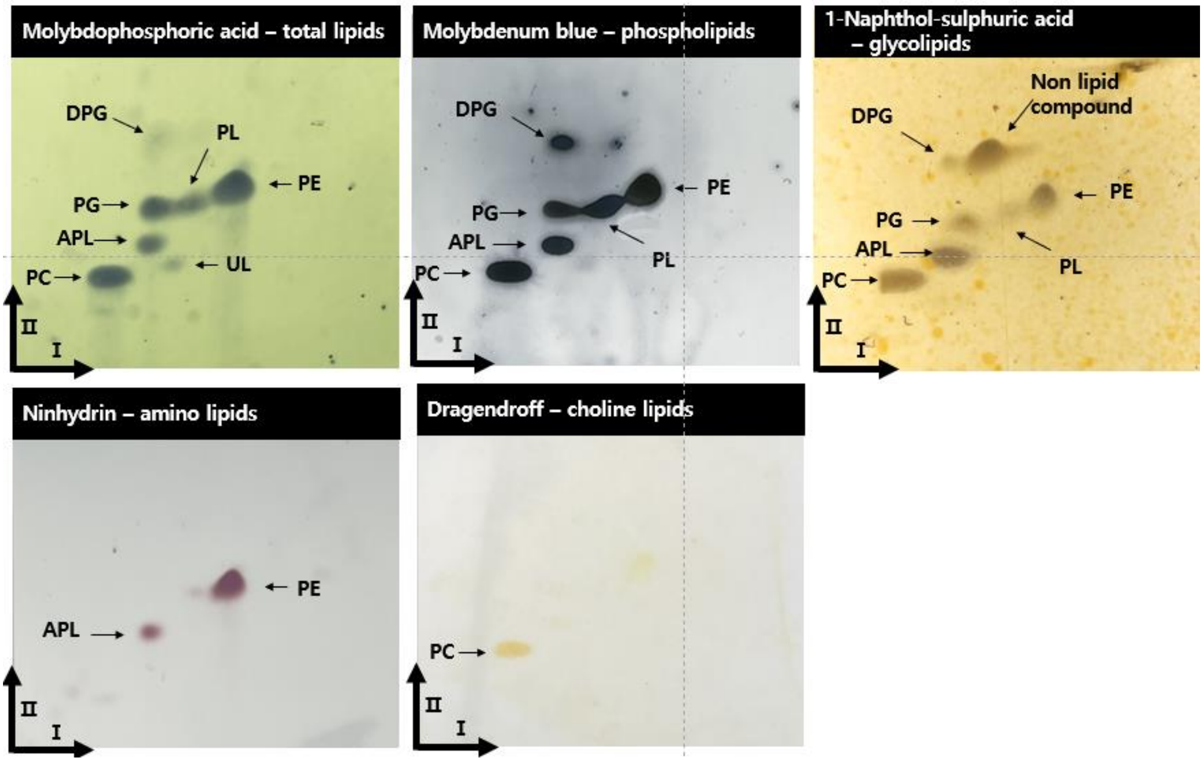

Figure S4. Two-dimensional thin-layer chromatograms showing the polar lipids of strain TT6^T. Solvent system: (I), chloroform/methanol/water (65:25:4, v/v/v); (II), chloroform/acetic acid/methanol/water 80:15:12:4, v/v/v/v). The spray agents and targeted lipids are indicated on plates. 1-Naphthol-sulphuric acid spray [1] did not give the expected violet-coloured spots for glycolipids. Abbreviations: PG, phosphatidylglycerol; PE, phosphatidylethanolamine; PC, phosphatidylcholine; DPG, diphosphatidylglycerol; PL, phospholipid; APL, aminophospholipid; PL, phospholipid; UL, unknown lipid.

Table S1. Gene information for the photosystem reaction center and pigment formation in strain TT6^T.

Locus_tag	Size/aa	Gene	Gene product
IGS68_17000	179	*bchF*	2-vinyl bacteriochlorophyllide hydratase
IGS68_17005	418	*bchN*	ferredoxin:protochlorophyllide reductase (ATP-dependent) subunit N
IGS68_17010	504	*bchB*	ferredoxin:protochlorophyllide reductase (ATP-dependent) subunit B
IGS68_17015	1230	*bchH*	magnesium chelatase subunit H
IGS68_17020	294	*bchL*	ferredoxin:protochlorophyllide reductase (ATP-dependent) iron-sulfur ATP-binding protein
IGS68_17025	229	*bchM*	magnesium protoporphyrin IX methyltransferase
IGS68_17030	474		BCD family MFS transporter
IGS68_17035	261	*pufA*	photosynthetic reaction center subunit H
IGS68_17040	230	*pufB*	PH domain-containing protein
IGS68_17045	152	*pufC*	hypothetical protein
IGS68_17050	99		hypothetical protein
IGS68_17055	351	*acsF*	magnesium-protoporphyrin IX monomethyl ester (oxidative) cyclase
IGS68_17060	272	*pufE*	DUF3623 domain-containing protein
IGS68_17065	406	*hemA*	5-aminolevulinate synthase
IGS68_17070	692		prolyl oligopeptidase family serine peptidase
IGS68_17075	67		type II toxin-antitoxin system VapB family antitoxin
IGS68_17080	387	*pufC*	photosynthetic reaction center cytochrome c subunit
IGS68_17085	325	*pufM*	photosynthetic reaction center subunit M
IGS68_17090	274	*pufL*	photosynthetic reaction center subunit L
IGS68_17095	63	*pufA*	light-harvesting complex 1 alpha chain
IGS68_17100	87	*pufB*	light-harvesting complex 1 beta chain
IGS68_17105	522	*bchZ*	chlorophyllide a reductase subunit Z
IGS68_17110	459	*bchY*	chlorophyllide a reductase subunit Y
IGS68_17115	328	*bchX*	chlorophyllide a reductase iron protein subunit X
IGS68_17120	311	*bchC*	chlorophyll synthesis pathway protein BchC
IGS68_17125	368		methyltransferase domain-containing protein
IGS68_17130	289		geranylgeranyl diphosphate synthase
IGS68_17135	518	*crtI*	phytoene desaturase
IGS68_17140	289	*crtC*	carotenoid 1,2-hydratase

Table S2. Gene information for the biosynthesis of bacteriochlorophylls in strain TT6^T.

Locus tag	Size/aa	Gene	Gene Product
IGS68_07670	405	*hemA*	5-aminolevulinate synthase
IGS68_23815	109	*hemB*	porphobilinogen synthase
IGS68_27285	314	*hemC*	hydroxymethylbilane synthase
IGS68_27290	257	*hemD*	uroporphyrinogen-III synthase
IGS68_26350	283	*hemE*	uroporphyrinogen decarboxylase
IGS68_04220	294	*hemF*	oxygen-dependent coproporphyrinogen oxidase
IGS68_10835	450	*hemN*	oxygen-independent coproporphyrinogen III oxidase
IGS68_26340	142	*hemJ*	protoporphyrinogen oxidase HemJ
IGS68_17195	581	*bchD*	magnesium chelatase subunit D
IGS68_17200	366	*bchI*	magnesium chelatase ATPase subunit I
IGS68_30880	548	*bchE*	magnesium-protoporphyrin IX monomethyl ester anaerobic oxidative cyclase
IGS68_07550	434		TIGR04295 family B12-binding domain-containing radical SAM protein
IGS68_30885	196	*bchJ*	bacteriochlorophyll 4-vinyl reductase
IGS68_17205	393	*chlP*	geranylgeranyl diphosphate reductase
IGS68_17210	294	*chlG*	chlorophyll synthase ChlG

Table S3. Gene information for the nitrogen fixation in strain TT6^{T}.

Locus tag	Size/aa	Gene	Gene product
IGS68_09220	659		ATP-binding response regulator
IGS68_09225	253	*yafJ*	Class II glutamine amidotransferase
IGS68_09230	130	nifO	Nitrogenase-associated protein NifO
IGS68_09235	297	*draG*	ADP-ribosyl-[dinitrogen reductase] hydrolase
IGS68_09240	287	*draT*	NAD(+)-dinitrogen-reductase ADP-D-ribosyltransferase
IGS68_09245	299	*nifH*	Nitrogenase iron protein
IGS68_09250	481	*nifD*	Nitrogenase molybdenum-iron protein alpha chain
IGS68_09255	376	*nifK*	Nitrogenase molybdenum-iron protein subunit beta
IGS68_09260	410		IS3 family transposase
IGS68_09265	131		Pseudogene (niK-3' end)
IGS68_09270	457	*nifE*	Nitrogenase iron-molybdenum cofactor biosynthesis protein NifE
IGS68_09275	456	*nifN*	Nitrogenase iron-molybdenum cofactor biosynthesis protein NifN
IGS68_09280	132	*nifX*	Nitrogen fixation protein NifX
IGS68_09285	152		NifX-associated nitrogen fixation protein
IGS68_09290	65		Hypothetical protein
IGS68_09295	97	*fdxB*	Ferredoxin III, nif-specific
IGS68_09300	188	*nifQ*	Nitrogen fixation protein NifQ
IGS68_09305	168		Hypothetical protein
IGS68_09310	132	*iscA*	Iron-sulfur cluster assembly accessory protein
IGS68_09315	316	nifU	Fe-S cluster assembly protein NifU
IGS68_09320	398	*nifS*	Cysteine desulfurase NifS
IGS68_09325	385	*nifV*	Homocitrate synthase
IGS68_09330	269	*cysE*	Serine O-acetyltransferase
IGS68_09335	111	nifW	Nitrogenase-stabilizing/protective protein NifW
IGS68_09340	276	*fixA*	Electron transfer flavoprotein subunit beta/FixA family protein
IGS68_09345	367	*fixB*	Electron transfer flavoprotein subunit alpha/FixB family protein
IGS68_09350	433	*fixC*	Flavoprotein-ubiquinone oxidoreductase
IGS68_09355	97	*fixX*	Ferredoxin family protein

Table S4. Gene information for the formation of hydrogenase complex in strain TT6^T.

Locus tag	Size/aa	Gene	Gene product
IGS68_07430	235	*hypX*	Crp/Fnr family transcriptional regulator
IGS68_07435	349	*hypE*	hydrogenase expression/formation protein HypE
IGS68_07440	381	*hypD*	hydrogenase formation protein HypD
IGS68_07445	89	*hypC*	HypC/HybG/HupF family hydrogenase formation chaperone
IGS68_07450	749	*hypF*	carbamoyltransferase HypF
IGS68_07455	303	*hypB*	hydrogenase nickel incorporation protein HypB
IGS68_07460	110	*hypA*	hydrogenase maturation nickel metallochaperone HypA
IGS68_07465	394		nickel-dependent hydrogenase large subunit
IGS68_07470	253	*hybE*	[NiFe]-hydrogenase assembly chaperone HybE
IGS68_07475	284		hydrogenase expression/formation protein
IGS68_07480	126		hypothetical protein
IGS68_07485	107		HypC/HybG/HupF family hydrogenase formation chaperone
IGS68_07490	203		HyaD/HybD family hydrogenase maturation endopeptidase
IGS68_07495	252	*cybH*	Ni/Fe-hydrogenase, b-type cytochrome subunit
IGS68_07500	597	*hupL*	nickel-dependent hydrogenase large subunit
IGS68_07505	366	*hupS*	hydrogenase small subunit

Table S5. Gene information for the oxidation of inorganic sulfur compounds in strain TT6^T.

Locus_tag	Size/aa	Gene	Gene product
IGS68_04765	136	*soxX*	sulfur oxidation c-type cytochrome SoxX
IGS68_04770	154	soxY	SoxY-related AACIE arm protein
IGS68_04775	106	soxZ	thiosulfate oxidation carrier complex protein SoxZ
IGS68_04780	217	*soxA*	sulfur oxidation c-type cytochrome SoxA
IGS68_23555	562	soxB	thiosulfohydrolase SoxB
IGS68_23560	109	soxZ	thiosulfate oxidation carrier complex protein SoxZ
IGS68_23565	159	soxY	thiosulfate oxidation carrier protein SoxY
IGS68_23590	445	*soxD*	sulfite dehydrogenase
IGS68_23595	434	*soxC*	cytochrome c

Table S6. Differential phenotypic results for strain TT6^T and related *Skermanella* type strains using the API 20E system.

Strains: 1, strain TT6^T; 2, *S. rosea* M1^T; 3, *S. mucosa* 8-14-6^T; 4, *S. pratensis* W17^T. All data are from this study. +, positive; −, negative; w, weakly positive.

	**1**	**2**	**3**	**4**
Nitrate reduction	−	+	+	+
β-Galactosidase (ONPG)	−	w	w	w
Arginine dihydrolase	−	−	−	−
Lysine decarboxylase	−	−	−	−
Ornithine decarboxylase	−	−	−	−
Citrate utilization	−	−	−	−
H_2S production	−	−	−	−
Urease	−	−	−	−
Tryptophan deaminase	−	−	−	−
Indole from L-tryptophan	−	−	−	−
Voges–Proskauer	w	−	+	+
Gelatinase	−	−	−	−
Fermentation/Oxidation				
D-Glucose	w	−	−	w
D-Mannitol	−	w	−	−
Inositol	−	−	−	−
D-Sorbitol	−	−	−	−
L-Rhamnose	w	−	−	w
D-Sucrose	−	−	−	−
D-Melibiose	−	−	−	−
Amygdalin	−	−	−	−
L-Arabinose	w	−	−	w

Table S7. Differential phenotypic results for strain TT6^T and related *Skermanella* type strains using the API 20NE system.

Strains: 1, strain TT6^T; 2, *S. rosea* M1^T; 3, *S. mucosa* 8-14-6^T; 4, *S. pratensis* W17^T. All data are from this study. +, positive; −, negative; w, weakly positive.

	**1**	**2**	**3**	**4**
Nitrate reduction	−	+	+	+
Indole from L-tryptophan	−	−	−	−
Arginine dihydrolase	−	−	−	−
Urease	−	−	−	−
β-Galactosidase (PNPG)	−	w	w	w
Hydrolysis of				
Esculin	w	w	w	w
Gelatinase	−	−	−	−
Fermentation				
D-Glucose	−	−	−	−
Assimilation				
D-Glucose	+	+	−	−
L-Arabinose	+	+	−	w
D-Mannose	w	+	−	−
D-Mannitol	+	+	−	−
N-Acetyl-glucosamine	−	−	−	−
D-Maltose	−	−	−	−
Potassium gluconate	+	+	−	+
Capric acid	−	−	−	−
Adipic acid	−	w	−	+
Malic acid	w	−	−	+
Trisodium citrate	−	−	−	+
Phenylacetic acid	−	−	−	−

Table S8. Differential phenotypic results for strain TT6^T and related *Skermanella* type strains using the API 50CH system.

Strains: 1, strain TT6^T; 2, *S. rosea* M1^T; 3, *S. mucosa* 8-14-6^T; 4, *S. pratensis* W17^T. All data are from this study. +, positive; −, negative; w, weakly positive.

	1	2	3	4
Control	−	−	−	−
Glycerol	−	−	−	+
Erythritol	−	−	−	+
D-Arabinose	−	+	−	+
L-Arabinose	+	+	+	+
D-Ribose	−	+	+	+
D-Xylose	+	+	+	+
L-Xylose	+	+	−	+
D-Adonitol	−	−	−	+
Methyl-β D-xylopyranoside	−	−	−	−
D-Galactose	−	+	−	+
D-Glucose	+	+	−	+
D-Fructose	+	+	−	+
D-Mannose	+	+	+	+
L-Sorbose	−	−	−	−
L-Rhamnose	+	−	−	+
Dulcitol	−	−	−	−
Inositol	−	−	−	−
D-Mannitol	+	+	+	−
D-Sorbitol	−	−	−	−
Methyl-α D-mannopyranoside	−	−	−	−
Methyl-α D-glucopyranoside	−	−	−	−
N-Acetylglucosamine	−	−	−	−
Amygdalin	−	−	−	−
Arbutin	−	−	−	−
Esculin (ferric citrate)	−	−	−	−
Salicin	−	−	−	−

D-Cellobiose	−	+	−	−
D-Maltose	−	−	+	+
D-Lactose (bovine origin)	+	+	+	−
D-Melibiose	−	−	+	−
D-Saccharose (sucrose)	−	−	+	−
D-Trehalose	−	−	+	+
Inulin	−	+	−	−
D-Melezitose	−	−	−	−
D-Raffinose	−	−	−	−
Amidon (starch)	−	−	+	−
Glycogen	−	−	−	−
Xylitol	−	−	−	−
Gentiobiose	+	−	−	−
D-Turanose	−	−	−	−
D-Lyxose	−	+	−	+
D-Tagatose	−	−	−	−
D-Fucose	+	+	+	+
L-Fucose	+	+	−	+
D-Arabitol	+	+	+	−
L-Arabitol	−	−	−	−
Potassium gluconate	+	+	−	+
Potassium 2-ketogluconate	+	−	−	+
Potassium 5-ketogluconate	−	−	−	+

Table S9. Differential phenotypic results for strain TT6^T and related *Skermanella* type strains using the API ZYM system.

Strains: 1, strain TT6^T; 2, *S. rosea* M1^T; 3, *S. mucosa* 8-14-6^T; 4, *S. pratensis* W17^T.

All data are from this study. +, positive; −, negative; w, weakly positive.

	**1**	**2**	**3**	**4**
Control	−	−	−	−
Alkaline phosphatase	+	+	+	+
Esterase (C4)	+	+	+	+
Esterase Lipase (C8)	+	+	+	+
Lipase (C14)	−	−	−	−
Leucine arylamidase	+	+	+	+
Valine arylamidase	+	+	+	+
Cystine arylamidase	w	w	w	w
Trypsin	−	−	−	−
α-Chymotrypsin	−	−	−	−
Acid phosphatase	w	w	+	−
Naphthol-AS-BI-phosphohydrolase	w	w	w	w
α-Galactosidase	−	−	−	−
β-Galactosidase	−	−	−	−
β-Glucuronidase	−	−	−	−
α-Glucosidase	+	w	w	+
β-Glucosidase	w	w	w	w
N-Acetyl-β-glucosaminidase	+	w	w	−
α-Mannosidase	−	−	−	−
α-Fucosidase	−	−	−	−

Table S10. Cellular fatty acid profiles of strain TT6^T compared with its closest phylogenetic relatives.

Strains; 1, strain TT6^T; 2, *S. rosea* M1^T; 3, *S. mucosa* 8-14-6^T; 4, *S. pratensis* W17^T. tr, trace (< 1.00%).

All data are from this study.

	**1**	**2**	**3**	**4**
**Fatty Acid**				
**Saturated**				
$C_{16:0}$	6.63	5.64	4.06	4.27
$C_{18:0}$	1.26	1.12	1.27	tr
**Unsaturated**				
$C_{16:1}$ ω11c	2.16	2.11	2.16	4.24
$C_{17:1}$ ω6c	1.57	tr	tr	tr
$C_{18:1}$ ω9c	1.75	1.91	2.56	2.75
**Hydroxy fatty acids**				
$C_{16:0}$ 3OH	3.30	2.60	2.47	3.40
$C_{18:1}$ 2OH	1.77	1.53	tr	2.31
**Summed feature**				
2; $C_{14:0}$ 3OH/$C_{16:1}$ iso I	5.12	4.64	4.13	6.18
3; $C_{16:1}$ ω7c/$C_{16:1}$ ω6c	1.51	1.64	1.33	1.83
8; $C_{18:1}$ ω7c/$C_{18:1}$ ω6c	73.53	76.58	79.30	70.85
